# Urbanization and Socioeconomic Disparities in Hypertension among Older Adult Women in Sudan

**DOI:** 10.5334/aogh.2404

**Published:** 2019-03-05

**Authors:** Shahd Osman, Christy Costanian, Nur Beyhan Annan, Fouad M. Fouad, Miran Jaffa, Abla M. Sibai

**Affiliations:** 1 Public Health Institute, Federal Ministry of Health, SD; 2 School of Epidemiology and Public Health, Faculty of Medicine, University of Ottawa, CA; 3 Department of Epidemiology and Population Health, Faculty of Health Sciences, American University of Beirut, LB

## Abstract

**Background:**

Evidence from the developed world associates higher prevalence of hypertension with lower socioeconomic status (SES). However, patterns of association are not as clear in Africa and other developing countries, with varying levels of socioeconomic development and epidemiological transition. Using wealth and education as indicators, we investigated association between SES and hypertension among older adult women in Sudan and examined whether urbanicity mediates the relationship.

**Methods:**

The sample included women aged 50 years and over participating in the nationally representative population-based second Sudan Health Household Survey (SHHS) conducted in 2010. Principal components analysis was used to assign each household with a wealth score based on assets owned. The score was categorized into quintiles from lowest (poorest) to highest (richest).

**Findings:**

The sample included a total of 5218 women, median and mean age 55 and 59 years, respectively, with the majority not have any schooling (81.6%). The overall prevalence of reported hypertension was found to be 10.5%. After adjustment for age, marital status, work status and urban/rural location, better wealth and higher education were independently and positively associated with hypertension prevalence rates. However, when stratified by urbanicity, the relationship between wealth and hypertension lost its significance for women in urban areas but maintained it in rural areas, increasing significantly and consistently with each increase in quintile index (adjusted odds ratio, aOR_1_ = 1.95 95% CI = 1.08–3.52; aOR_2_ = 5.25, 95% CI = 3.01–9.15; aOR_3_ = 8.27, 95% CI = 4.78–14.3; and aOR_4_ = and 11.4, 95% CI = 6.45–20.0; respectively). By contrast, education played a greater role in increasing the odds of hypertension among women in urban locations but not in rural locations (aOR = 2.14, 95% CI = 1.25–7.90 vs. aOR = 0.79, 95% CI = 0.27–2.30, respectively).

**Conclusions:**

Our findings of a socioeconomic gradient in the prevalence of hypertension among women, mediated by urbanization, call for targeted interventions from early stages of economic development in Sudan and similar settings of transitioning countries.

## Introduction

Hypertension is a leading global public health problem and is an established risk factor for cardiovascular diseases, kidney conditions, cognitive decline and other non-communicable diseases (NCDs) [1,2]. Evidence suggests that hypertension-related burden of disease is especially striking in low- and middle-income countries (LMICS), with the highest rates being reported in Africa [3]. The increase in the prevalence of hypertension in LMICs in recent years has been attributed to rapid urbanization and associated societal and environmental changes in lifestyle, and demographic changes with a growing number of ageing adults [4,5,6]. The trend of increasing prevalence of chronic disease will continue in lower income countries, exerting a significant burden on the individual, society and health systems, already overburdened with limited resources.

The Republic of Sudan is a low-income country and the third largest in the African continent. Driven by agriculture and supported by improved macroeconomic policies, Sudan’s economic growth rose to above 5% in 2015 and is expected to increase further in the near future. Yet, economic stability and the consequences of the civil war that ravaged the country from 1983 to 2005 remain the most pressing challenges. Although the country is rich in resources, their availability is greatly skewed and mostly untapped to the extent that almost half of the Sudanese are classified as poor, with considerable inequities across and within states [7,8].

Studies on hypertension prevalence rate in Sudan remain sparse, with variations in findings depending on age of participants, gender mix, definition and measurement of hypertension, and study setting (rural/urban, or national/sub-national). For example, statistics from the Federal Ministry of Health indicate that the registered number of hypertension cases constitute around 10% of the total population [9]. The WHO stepwise survey conducted in 2005 gave a prevalence rate of reported hypertension of 11.3% among adults aged 25–64 years in Khartoum, the capital city [10], and a 15% estimate was obtained from a study in four states in rural Sudan [11]. Furthermore, little is known on disparities between rural and urban areas or by socioeconomic status such as educational level or household wealth. Evidence from developed countries associates higher socioeconomic status (SES) with an overall lower prevalence of high blood pressure and cardiovascular disease [12,13], with findings being most evident for women [14,15]. By contrast, the pattern of association is not as clear in Africa and other developing countries, where a mix of positive and negative gradients has been found across studies and along a country’s socioeconomic development and epidemiological transition [16,17,18,19]. With an unbalanced urban-rural development structure in Sudan, patterns of economic growth and poverty vary tremendously across and within regions [20]. This study aimed to determine socioeconomic disparities associated with hypertension in Sudan, focusing attention to older women, an under-researched group in the region. We hypothesized that measures of SES, including education and wealth, would be positively and independently associated with hypertension among older adult Sudanese women, and that these associations would vary by urbanicity, thus the participants’ rural/urban area of dwelling acting as effect modifier.

## Methods

### Study design and participants

Data for this study were drawn from the nationally representative population-based second Sudan Health Household Survey (SHHS), conducted by the Federal Ministry of Health and the Central Bureau of Statistics between March and May 2010 [21]. The SHHS sample was selected using two-stage cluster sampling with probability proportional to size targeting each of the, back then, 15 States of Sudan. Within each state, 40 census enumeration areas (EA) were selected, and 25 households were drawn randomly from each EA, including nomadic households. Out of 15,000 targeted households, 14,778 had complete interviews (98.5%). The Household Roster identified a total of 18,614 women aged 15 years and over, of which 17,174 were interviewed (response rate 92.3%). With a focus on older women in this study, the data were restricted to those aged 50 years and above, yielding 5218 participants available for the analytical sample.

Interview schedules were developed by the global Multiple Indicator Cluster Survey (MICS) project and aimed at providing up-to-date information on the situation of women in Sudan targeting achievements towards the Millennium Development Goals. It consisted of five sets of questionnaire modules. In this analysis, we focus on the Household Questionnaire module that included information on demographic and socioeconomic indicators and selected chronic diseases including hypertension for all de jure household members. Ethical permission for the SHHS was obtained from the National Ethical Review Committee, Federal Ministry of Health, Khartoum, Sudan. The data for the present study were obtained with permission from the Central Bureau of Statistics, Khartoum Sudan (www.cbs.gov.sd).

### Measures

Hypertension, the main outcome variable, was ascertained if a participant replied positively to the option hypertension from a list of 15 chronic conditions following the question: “Which of the following conditions do you suffer from?” with responses being coded as absent/present. SES variables included education and wealth index. Levels of education included ‘no school’, ‘primary’ ‘khalwa’, ‘adult learning’, and ‘secondary and above’. Primary education, khalwa, and adult learning were later grouped together into ‘primary education’ as these represent comparable levels of education. For measuring wealth, a composite measure of the household’s living standards and assets was computed using data from the Household Questionnaire that tapped on household’s ownership of selected belongings with the purpose of capturing household affluence. Using principal components analysis, each household was assigned a wealth score based on assets owned including ownership of land for farming, fishing or grazing, consumer goods, dwelling characteristics, water and sanitation, among others. The final score of the wealth index ranged from (–1.2 to 3.7), with those in the minus category representing below the poverty line in Sudan. The score was then divided into quintiles from lowest (poorest, –1.2 to –0.9) to highest (richest, 1.1 to 3.7). Further details on the construction of the wealth index for this survey can be found elsewhere [22]. Other covariates were assessed in relation to hypertension, and these included: age (‘50–54’, ‘55–59’, ‘60–64’, and ‘≥65’ years), marital status (‘never married’, ‘married’, ‘widowed/divorced/separated’), employment (‘employed’, ‘not employed’), and urban/rural dwelling. Urban/rural classification of areas in Sudan is defined by national statistical offices and is routinely reported in all national surveys.

### Statistical Analysis

**Figure 1:**
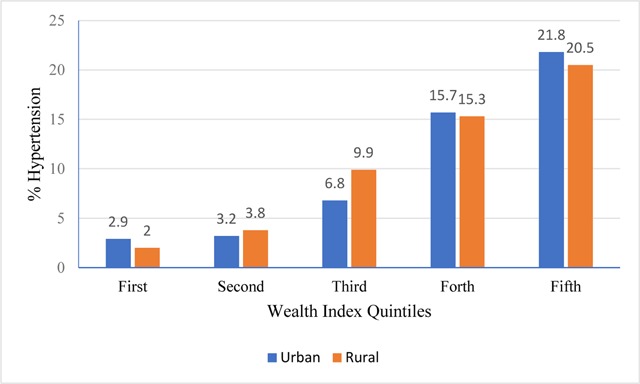
Prevalence rates of hypertension among older adult Sudanese women by wealth index and urban/rural areas of residence (n = 5218), Sudan Health Household Survey, 2010. Sample size 1744 for urban and 474 for rural.

Descriptive statistics expressed as frequencies and percentages were generated to describe the participant characteristics. The prevalence of hypertension was estimated in the total sample and by baseline attributes, and a chi-square test being used to examine differentials in hypertension by demographic and socio-economic characteristics (Table 1). Additionally, the analysis was stratified by area of dwelling (urban/rural) for the wealth index (Figure 1). The association between various covariates and the wealth index was then examined to check for potential confounding variables (Table 2). Finally, multiple regression analyses were conducted in the total sample and stratified by urban/rural dwelling, treated in our analyses as an effect modifier, while controlling for covariates. Adjusted odds ratios (aORs) and their 95% CIs were estimated (Table 3). Significance level was set at 0.05 and the SPSS version 22.0 software package was used for all analyses.

**Table 1:** Distribution of baseline characteristics in the total population and by hypertension status in older adult Sudanese women, SHHS, 2010.

Variables	Total		Non-Hypertensive		Hypertensive		*p*-value

	N	%	n	%	n	%	

Total Sample	5218*	100	4670*	89.5	548*	10.5	

**Age (years)**							
50–54	2180	42.0	2020	92.7	160	7.3	<0.001
55–59	861	16.6	772	89.7	89	10.3	
60–64	761	14.7	657	86.3	104	13.7	
≥65	1388	26.7	1193	86.0	195	14.0	
**Education**							
No school	4257	81.6	3871	90.9	386	9.1	<0.001
Primary ^†^	830	15.9	697	84.0	133	16.0	
Secondary	130	2.5	101	77.7	29	22.3	
**Work status**							
Not working	4201	81.3	3738	89.0	463	11.0	0.009
Working	966	18.7	887	91.8	79	8.2	
**Marital Status**							
Never married	110	2.1	102	92.7	8	7.3	0.433
Married	3035	58.3	2721	89.7	314	10.3	
Divorced/widowed	2061	39.6	1836	89.1	225	10.9	
**Area**							
Rural	3474	66.6	3183	91.6	291	8.4	<0.001
Urban	1744	33.4	1487	85.3	257	14.7	
**Wealth index quintiles**							
First (low)	817	15.7	800	97.9	17	2.1	<0.001
Second	1156	22.2	1113	96.3	43	3.7	
Third	1125	21.6	1027	91.3	98	8.7	
Fourth	1067	20.4	902	84.5	165	15.5	
Fifth (high)	1053	20.2	828	78.6	225	21.4	

* The subtotals for each variable do not necessarily add to the grand total because of missing data. ^†^ This includes in addition to primary education, khalwa and adult learning.

## Results

Table 1 presents the baseline characteristics of women included in the present analysis. The sample was skewed towards younger age groups, with the majority (42.0%) being in the age bracket between 50 and 54 years (median and mean age [SD], 55, 59 [10] years, respectively). Four out of five women in the sample did not have any schooling (81.6%), a similar proportion were not working at the time of the survey (81.3%), 58.3% were currently married and 39.6% were widowed/divorced. The proportion of participants living in rural areas was almost two-fold those living in urban areas (66.6% and 33.4%, respectively). Except for the lowest wealth index quintile (15.7%), the study sample was equally distributed across the remaining quintiles (20.2%–22.2%).

The overall prevalence of reported hypertension was found to be 10.5%. It increased consistently with age reaching 14.0% among older women aged 65 years and over (Table 1). Although there was no significant variation by marital status, hypertension prevalence was significantly higher among those with secondary schooling compared to no or little schooling (22.3% vs. 9.1–16.0%, respectively) and among the not working compared to the working population (11.0% vs. 8.2%). The rate of hypertension was significantly higher in urban area compared to the rural (14.7% and 8.4%, respectively).

Rates of hypertension differed by wealth index quintiles, increasing consistently with increasing wealth in the total sample (Table 1) and in both urban and rural areas (Figure 1). Women in the highest wealth quintile were 10 times more likely to report hypertension compared to those in the lowest (21.4% vs. 2.1%). Table 2 illustrates the associations between the wealth index and other covariates. Compared to their counterparts, women with higher education, non-working, the never married and those living in urban areas were significantly more likely to be living in households characterized by higher wealth index.

**Table 2:** Association between co-variates and the wealth index, SHHS, 2010.

	Wealth Index quintiles															*p*-value

	First (low)			Second			Third			Forth			Fifth (high)			
		
	n		%	n		%	n		%	n		%	n		%	

**Mean wealth score** ± **SD**	–1.03	±	0.08	–0.71	±	0.12	–0.13	±	0.22	0.70	±	0.26	1.82	±	0.56	

**Age (years)**																
50–54	329		15.1	524		24.0	479		22.0	437		20.0	411		18.9	0.152
55–59	136		15.8	194		22.5	178		20.7	175		20.3	178		20.7	
60–64	136		17.9	147		19.3	168		22.1	149		19.6	161		21.2	
≥65	210		15.1	284		20.5	295		21.3	297		21.4	302		21.8	
**Education**																

No school	789		18.5	1097		25.8	972		22.8	807		19.0	592		13.9	<0.001
Primary*	28		3.4	59		7.1	146		17.6	242		29.2	355		42.8	
Secondary	0			0			7		5.4	18		13.8	105		80.8	
**Work status**																

Working	237		24.5	270		28.0	189		19.6	130		13.5	140		14.5	<0.001
Not working	574		13.7	865		20.6	925		22.0	930		22.1	907		21.6	
**Marital Status**																
Never married (single)	13		11.8	15		13.6	24		21.8	27		24.5	31		28.2	<0.001
Married	472		15.6	651		21.4	630		20.8	619		20.4	663		21.8	
Divorced/widowed	332		16.1	486		23.6	466		22.6	418		20.3	359		17.4	
**Area**																
Rural	783		22.5	1031		29.7	698		20.1	582		16.8	380		10.9	<0.001
Urban	34		1.9	125		7.2	427		24.5	485		27.8	673		38.6	

To determine relative importance of wealth index versus education, we performed a stepwise multivariate logistic regression stratified by urban/rural locations (Table 3). The odds of having hypertension in the total sample of women increased consistently with increasing wealth index, with a significant dose response relationship (p-value for trend < 0.05). Upon stratification by area of residence, the relationship between hypertension and wealth index mostly lost its significance in the urban area but maintained it in the rural area, increasing consistently with each increase in quintile index (from first to fifth) (aOR_1_ = 1.95 95% CI = 1.08–3.52; aOR_2_ = 5.25, 95% CI = 3.01–9.15; aOR_3_ = 8.27, 95% CI = 4.78–14.3; and aOR_4_ = and 11.4, 95% CI = 6.45–20.0; respectively). By contrast, secondary education played a significantly greater role increasing the odds of hypertension among women in urban locations than in rural locations (aOR = 2.14, 95% CI = 1.25–7.90 vs. aOR = 0.79, 95% CI = 0.27–2.30, respectively).

**Table 3:** Associations between wealth index and hypertension in the total sample and by urban/rural area of the dwelling, SHHS, 2010.

	Total sample		Rural		Urban	

	Adjusted OR*	95% CI	Adjusted OR^†^	95% CI	Adjusted OR^†^	95% CI

**Wealth Index**						
First (low)	1.00	Reference	1.00	Reference	1.00	Reference
Second	1.89	1.07–3.35	1.95	1.08–3.52	1.16	0.13–10.8
Third	4.52	2.67–7.67	5.25	3.01–9.15	2.35	0.31–17.9
Forth	8.33	4.97–13.9	8.27	4.78–14.3	5.68	0.76–42.4
Fifth (high)	11.4	6.73–19.2	11.4	6.45–20.0	7.50	1.01–55.8
**Education**						
No school	1.00	Reference	1.00	Reference	1.00	Reference
Primary	1.39	1.09–1.76	1.42	0.99–2.01	1.36	0.98–9.23
Secondary	1.76	1.11–2.78	0.79	0.27–2.30	2.14	1.25–7.90

* OR is adjusted for age, education, marital status, and urban/rural area of living. ^†^ OR is adjusted for age, education, marital status.

## Discussion

Findings from this nationally representative study show a relatively low prevalence of reported hypertension among adult Sudanese women aged 50 years and older. Significant disparities in prevalence rates were noted with socio-economic indicators, including education and household wealth. Compared with their counterparts, hypertension prevalence rates were higher among the more educated and in households with higher wealth index. The relationship with household wealth, however, varied by urbanicity, being significant in rural but not in urban areas. These findings are discussed below within the context of socio-economic conditions and epidemiologic transition Sudan is currently passing through.

Close to 11% of older adult women in Sudan reported a history of hypertension. This appears to be comparable to results from a similar study of a large nationally representative sample of women in Ghana (11.4%, computed from data for women aged 40 years and over) [23] but lower than worldwide and regional estimates. In an earlier systematic review on hypertension rates in Sub-Saharan Africa, prevalence rates ranged between 6% to 48% [24]. Hypertension is a silent disease and many cases remain undiagnosed. The prevalence of hypertension in this study was based on self-reports, and there were no questions about medications use or validation of hypertension reports. Also, some respondents may have been unaware of their conditions and/or diagnosis have been made but not conveyed properly to the patient. Rates of unawareness vary widely in the literature, with LMIC countries having approximately twice the proportions of hypertension unawareness in comparison to high-income countries (62% vs. 33%) [25]. While the proportion of hypertension unawareness has been decreasing in the recent past in the western world, it remains problematic in low-income countries. In these settings, improving awareness and timely identification of hypertension are the most important targets for intervention and key for the control of hypertension.

Our finding of overall greater prevalence of hypertension among women in urban locations compared to the rural is in accordance with studies from other countries in Africa and south Asia [26,27,28]. Urbanization is accompanied by transition in the environments that impact on food choices and behaviors, with greater likelihood of adoption of western style diets and sedentary lifestyles leading to increased prevalence of cardiometabolic risks [29,30]. Urbanization is also tagged with development of social infrastructure, significant economic transformations, increased spending on education and healthcare and hence better health awareness and enhanced hypertension diagnosis [30].

We also found overall greater prevalence of hypertension among women with greater wealth. Whilst associations of hypertension with urbanity was attenuated and became nonsignificant in both the stratified and multivariate analysis, differentials by wealth remained significant and was evident in rural areas but not urban areas. The role of SES and wealth in hypertension varies across settings and depends on the timing and stage of the epidemiological transition. In developed countries, a higher prevalence of cardiovascular risk factors is seen among individuals with low SES living conditions [29]. This association does not necessarily hold in developing countries with recent economic and epidemiologic transitions and increase in over nutrition and sedentary lifestyles with increasing economic resources and wealth [31]. In a recent study, Elhuda and colleagues [32] have shown higher odds of hypertension among a sample of 255 women in rural Sudan with higher socioeconomic status and lower literacy compared to their counterparts. Similarly, the prevalence of hypertension in Jamaica was highest in the wealthiest women and those with better housing asset index compared with poorer women [33] and among women from low-income rural populations in Mexico [16]. The observed worsening in hypertension with upward mobility in wealth in rural Sudan follows Ezzati et al.’s reflection that the transition from low cardiovascular risk to high-risk in low socioeconomic individuals follows the gross national income, and only when the gross national income of a country exceeds a certain level does the inversion of risk factors take place [34].

There are limited studies in low and low-middle income countries that have examined association of socioeconomic factors such as household wealth, educational status, and other social factors with hypertension [35]. Rapid urbanization and the lack of an adequate infrastructure influence the creation of large socioeconomic disparities, which also affect health outcomes, including hypertension [29]. Yet, the theory of ‘social transition’ of NCD risk factors from the higher to the lower socioeconomic groups with the country’s development remains debated [19], with inconsistencies in the findings owing to variations in the SES indicators used and the population mix. A comprehensive understanding of the relationship between SES and hypertension requires also information on associations with unique measures of household wealth at various points in time, that may better reflect access to resources and command. This may be particularly of significance in low-income countries with various urban-rural settings and resources and with different levels of development.

The study findings need to be interpreted considering certain limitations and offsetting strengths. Hypertension comes without symptoms especially in the early stages, and this is likely to introduce information bias resulting in an underestimate of prevalence rates. This, however, may be less of a problem in middle-aged and older persons who have greater opportunities to be seen by the healthcare system and hence diagnosed than the young. It is possible that the less educated women in our study and those in rural areas with limited access and availability of health care were more unaware of their hypertension than their counterparts, hence the lack of associations between education and hypertension among rural older women. Furthermore, the use of secondary data precluded us from examining associations with other micro-level social determinants of health and other cardio-metabolic factors, such as access to healthcare and diabetes, as these were lacking in the original questionnaire. Yet, the large sample size and the use of international guidelines and standardized protocol for sampling, interview schedule, and field methodology (MICS) enable generalizability of our results to all of Sudan with findings serving as a benchmark for future assessments in the country and allowing for comparison with similar studies in the region.

## Conclusion

As elsewhere in emerging economies, the epidemiological transition in Sudan from predominantly infectious to chronic diseases is already underway. Yet studies on NCDs and metabolic diseases have scarcely begun to be explored. Once linked to be diseases exclusively of the wealthy, NCDs will continue to be a burden on the world’s poor. An examination of socioeconomic disparities in hypertension in settings of persistent burden of infectious diseases can provide important clues for defining at-risk populations and for designing context-specific and appropriate primary prevention and treatment programmes. For a better understanding of the social epidemiology of hypertension, repeated surveys will be needed to allow better monitoring of the complex trajectories in exposures and changes in risk factor associations across time in Sudan and in similar settings elsewhere.
